# Siloxane-Reinforced GelMA/Methacrylated Chitosan Hydrogels for DLP Printing

**DOI:** 10.3390/gels12090833

**Published:** 2026-09-11

**Authors:** Yufan Zhang, Jing Wang, Bo Liu, Xueqin Zhang, Yunxuan Weng

**Affiliations:** 1 School of Advanced Materials and Future Technology, Beijing Technology and Business University, Beijing 102488, China; 2 Plastic Beijing Municipal Key Laboratory of Health and Safety Quality Evaluation Technology, Beijing 100048, China; 3 College of Mathematics and Physics, Beijing University of Chemical Technology, Beijing 100029, China

**Keywords:** GelMA, CSMA, MPTS-derived siloxanes, composite hydrogels, photopolymerization, DLP fabrication

## Abstract

Photocrosslinked gelatin methacryloyl/methacrylated chitosan (GelMA/CSMA) hydrogels are attractive for biomedical applications but are limited by insufficient mechanical strength. Here, γ-methacryloxypropyltrimethoxysilane (MPTS) was prehydrolyzed and condensed to generate siloxane species retaining polymerizable methacrylate groups, which were subsequently incorporated into the GelMA/CSMA network as a reinforcing phase. The effects of MPTS precursor concentration (2–5% *w*/*v*) and prehydrolysis time (12–48 h) on the morphology, viscoelasticity, compressive behavior, cytocompatibility, and DLP printability of the resulting GCM hydrogels were systematically investigated. FTIR confirmed methoxy-group consumption while retaining C=C functionality. SEM showed that increasing MPTS precursor concentration increased the apparent abundance of granular features, whereas prolonged prehydrolysis produced larger structures. XRD indicated a predominantly amorphous MPTS-derived phase. GCM-48 h-5% achieved a storage modulus of 23.5 kPa, compressive modulus of 0.682 MPa, and maximum compressive stress of 1.081 MPa, corresponding to 7.4-, 16.9-, and 4.0-fold increases over the GelMA/CSMA control, respectively. The hydrogels exhibited favorable L929 cytocompatibility and retained DLP printability under 405 nm irradiation. These findings demonstrate that regulating MPTS precursor concentration and prehydrolysis time provides a simple route to mechanically reinforce DLP-printable GelMA/CSMA hydrogels without directly incorporating preformed silica particles.

## 1. Introduction

Bone defects caused by trauma, tumor resection, congenital abnormalities, and degenerative diseases remain a major clinical challenge [1]. Tissue-engineered scaffolds are therefore expected to provide a hydrated environment favorable for cellular activity while maintaining sufficient mechanical performance and structural integrity [2,3]. Hydrogels are attractive scaffold materials because their water-rich three-dimensional networks resemble important features of the native extracellular matrix, support cell adhesion and migration, and facilitate molecular transport [4,5]. To translate these biological advantages into scaffolds with customized geometries and well-defined internal architectures, precise fabrication techniques are required. Among these, digital light processing (DLP) has emerged as a powerful tool due to its high resolution and rapid fabrication capabilities [4,6,7,8]. The hydrogel formulation must therefore support reliable DLP printing while providing the printed scaffold with sufficient mechanical stability and favorable biological performance.

Gelatin methacryloyl (GelMA) is widely used in DLP biofabrication because it retains cell-interactive and enzymatically degradable sequences from gelatin while allowing rapid network formation through methacrylate-mediated photopolymerization [9,10,11,12]. Methacrylated chitosan (CSMA) provides a complementary component because of its biocompatibility, biodegradability, abundant functional groups, and potential antimicrobial activity [13,14,15,16]. Upon photocrosslinking, GelMA and CSMA form an integrated covalently crosslinked network whose mechanical properties can be tuned by varying their relative proportions [17,18]. Increasing polymer concentration or photocrosslinking density can improve stiffness [19]. However, higher polymer content increases the viscosity of the printing precursor and may hinder resin flow and layer replenishment during DLP printing, whereas excessive crosslinking can restrict network hydration, molecular transport, degradation, and cellular activity [19,20]. Introducing a dispersed reinforcing phase therefore provides an alternative strategy to enhance mechanical performance while preserving DLP processability and cytocompatibility.

Silica-based particles [21,22], bioactive glasses [23], and calcium phosphate ceramics [1] have been investigated as reinforcing phases in hydrogel composites [24]. Among them, silica-based materials are particularly attractive because their relatively rigid structures can contribute to load transfer, while surface silanol groups can interact with hydroxyl, amino, and amide groups in gelatin- and chitosan-based matrices. Such interfacial interactions, together with filler content and spatial distribution, strongly influence the mechanical response of reinforced hydrogels [25,26]. However, conventional approaches generally rely on the direct incorporation of preformed particles into polymer precursor solutions. In complex aqueous formulations, these particles may aggregate or sediment, reducing effective interfacial interactions and producing heterogeneous reinforcement. In DLP systems, suspended particles and their aggregates can alter light propagation through scattering and attenuation, thereby affecting the cure depth, lateral polymerization, and ultimately the dimensional fidelity of printed structures [27,28]. These limitations complicate the simultaneous optimization of mechanical reinforcement, dispersion stability, and DLP processability.

γ-Methacryloxypropyltrimethoxysilane (MPTS) offers an alternative route as it possesses both hydrolyzable trimethoxysilane groups and a polymerizable methacrylate group [29]. Hydrolysis and condensation of MPTS generate siloxane-rich structures, while the retained methacrylate functionality participates in subsequent photopolymerization with GelMA and CSMA. This prehydrolysis–condensation–photocrosslinking strategy therefore directly couples the formation of an MPTS-derived reinforcing phase with the construction of the polymer network, bypassing the need for preformed silica particles. Previous studies have reinforced photocurable GelMA-based hydrogels by incorporating preformed silica particles, silanized silica, silanized hydroxyapatite, bioactive glass, or acrylate-functionalized nano-silica into the polymer matrix [30,31]. These approaches demonstrate that inorganic reinforcement and silane-mediated interfacial coupling can effectively improve the mechanical performance of photocrosslinked hydrogels. In contrast, the present study uses MPTS itself as a molecular precursor, which undergoes controlled prehydrolysis and condensation before incorporation into the GelMA/CSMA system. The retained methacrylate functionality further allows the resulting MPTS-derived species to participate in subsequent photocrosslinking. Thus, the reinforcing siloxane structures are generated from a molecular silane precursor rather than introduced as separately prepared inorganic particles.

In this study, MPTS concentration and prehydrolysis time were systematically varied to construct MPTS-reinforced GelMA/CSMA composite hydrogels. The evolution of MPTS during prehydrolysis and the resulting MPTS-derived structures were characterized using Fourier-transform infrared spectroscopy, scanning electron microscopy, and X-ray diffraction. Viscoelastic behavior, compressive properties, and 24 h swelling were evaluated to establish relationships among processing conditions, structural characteristics, and macroscopic performance. Cytocompatibility toward L929 fibroblasts and DLP printability under 405 nm irradiation were further assessed. By integrating controlled MPTS prehydrolysis with photocrosslinking of methacrylated biopolymers, this study provides a simple strategy for improving the mechanical performance of DLP-processable GelMA/CSMA hydrogels while avoiding direct incorporation of preformed reinforcing particles.

## 2. Results and Discussion

### 2.1. Chemical Structure Characterization

**Figure 1:**
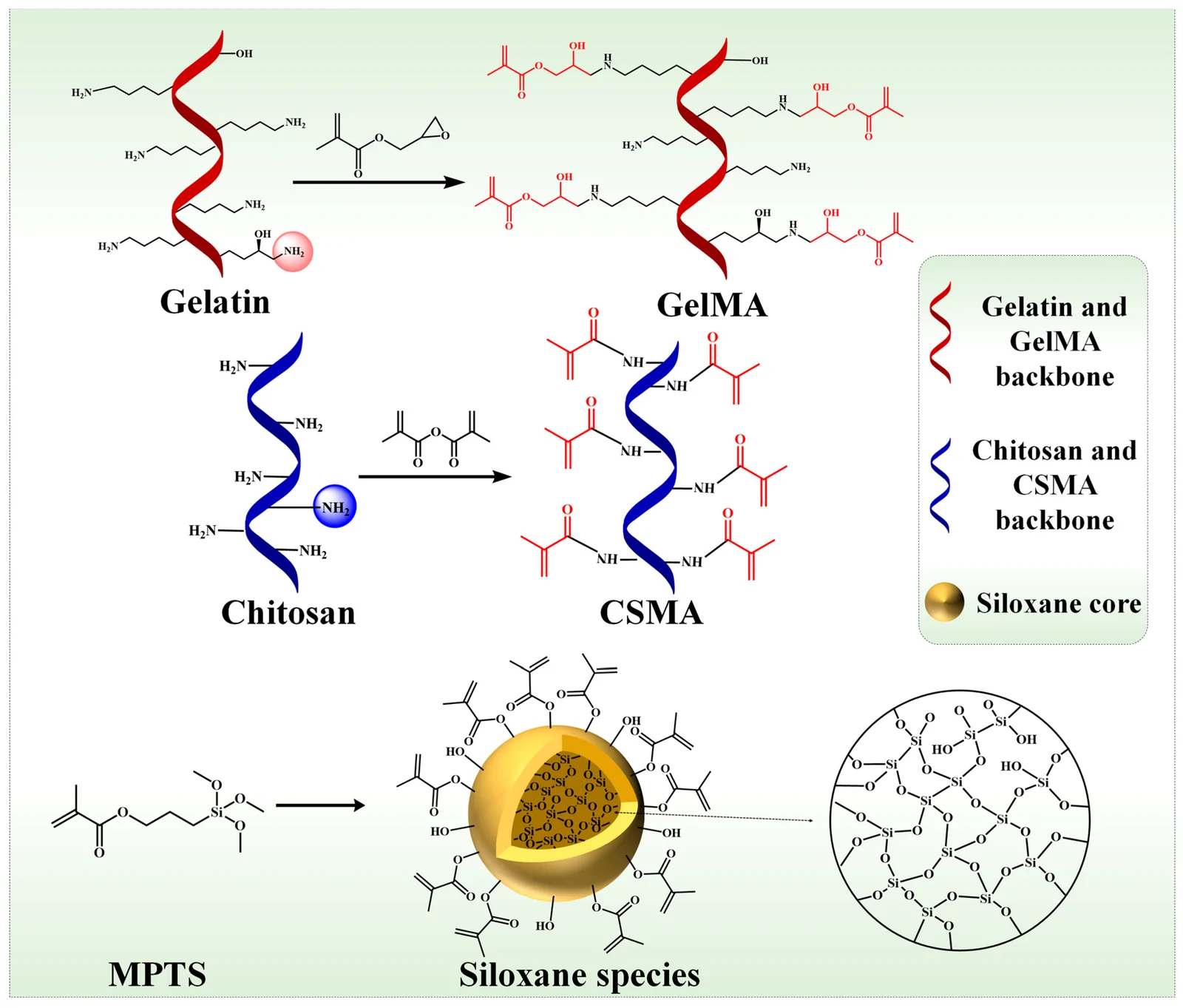
Schematic illustration of the synthesis of GelMA and CSMA and the prehydrolysis–condensation of MPTS to form siloxane species.

Polymerizable methacryloyl groups were grafted onto gelatin and chitosan backbones using glycidyl methacrylate (GMA) in PBS and methacrylic anhydride (MAA) in aqueous acetic acid, respectively (Figure 1). Siloxane species were synthesized through the hydrolysis and condensation of MPTS under mild conditions via a sol–gel process (Figure 1).

**Figure 2:**
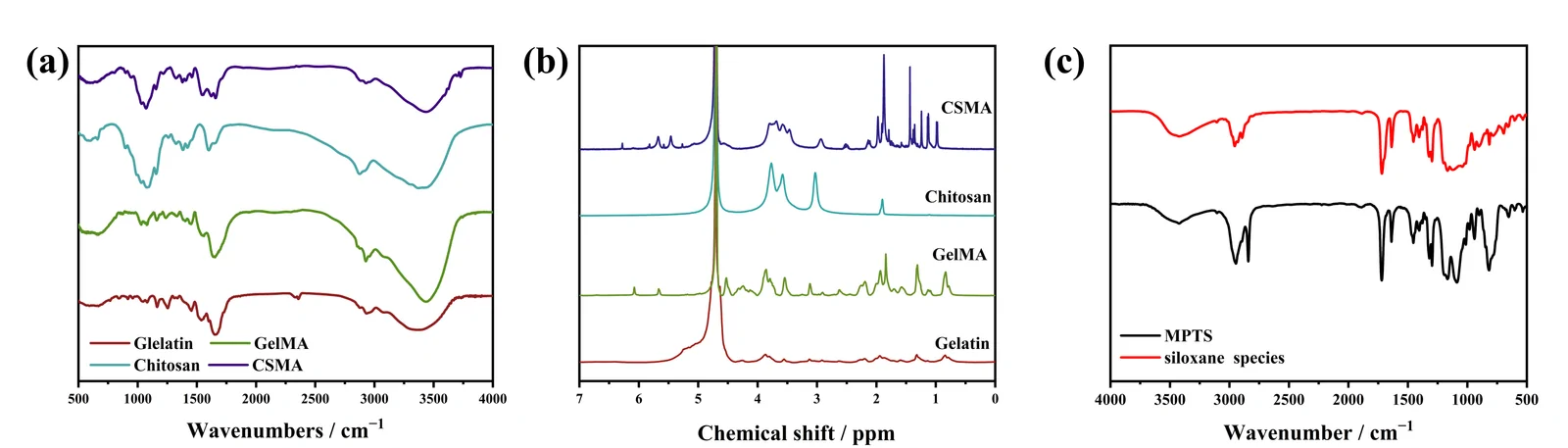
Chemical characterization of the synthesized materials: (**a**) FTIR spectra of gelatin, GelMA, chitosan, and CSMA; (**b**) ^1^H NMR spectra of gelatin, GelMA, chitosan, and CSMA; and (**c**) FTIR spectra of MPTS before and after 48 h prehydrolysis.

Figure 2a compares the FTIR spectra of GelMA and CSMA with those of gelatin and chitosan, respectively. Gelatin exhibited characteristic amide bands of proteins, including amide I at 1655 cm^−1^, assigned to C=O stretching; amide II at 1534 cm^−1^, associated with N–H bending coupled with C–N stretching; and amide III at approximately 1248 cm^−1^, attributed to C–N stretching and N–H bending. The peaks at 2936 and 2872 cm^−1^ corresponded to the asymmetric and symmetric stretching vibrations of aliphatic C–H groups, respectively, while the broad band near 3400 cm^−1^ was assigned to N–H and O–H stretching. GelMA retained the characteristic amide bands of gelatin, indicating preservation of the protein backbone. Changes in the positions and shapes of the amide I and II bands reflected alteration of the local chemical environment after methacryloyl grafting [32,33]. Meanwhile, a new absorption peak appeared at 1638 cm^−1^, assigned to the C=C stretching vibration, providing direct evidence for the presence of the polymerizable double bonds.

Chitosan exhibited characteristic bands at approximately 1026 cm^−1^ and 1155 cm^−1^, assigned to asymmetric C–O–C stretching of the glycosidic linkage and C–O stretching of the polysaccharide backbone, respectively (Figure 2a). The N–H bending vibration of primary amino groups appeared near 1597 cm^−1^, while the bands between 2800 and 2975 cm^−1^ were attributed to aliphatic C–H stretching. The broad peak at approximately 3400 cm^−1^ was attributed to the O–H and N–H stretching vibrations. After methacryloyl modification, CSMA showed an enhanced carbonyl absorption near 1662 cm^−1^, which was attributed to the contribution of newly introduced amide groups. A band near 1620 cm^−1^ was consistent with the presence of methacrylate C=C groups, while the N–H bending band shifted from 1597 to 1548 cm^−1^, indicating a change in the chemical environment of the amino groups. In addition, the absorption intensity of the amide III band at 1319 cm^−1^ increased slightly [34,35].

The ^1^H NMR further confirmed the successful modification of gelatin and chitosan. As shown in Figure 2b, GelMA exhibited new signals at 6.08 and 5.66 ppm, assigned to the vinyl protons of the methacryloyl groups [33]. Similarly, CSMA showed characteristic vinyl proton signals at 5.67 ppm and 5.46 ppm [36]. These results confirm the successful introduction of polymerizable methacryloyl groups.

Figure 2c compares the FTIR spectra of MPTS before and after hydrolysis. The characteristic C=O and C=C stretching bands remained at approximately 1721 and 1638 cm^−1^, respectively. Notably, these bands were retained after hydrolysis, indicating that the methacrylate group was preserved during the sol–gel process. In contrast, the methoxy-related C–H absorption near 2840 cm^−1^ decreased after hydrolysis, consistent with the conversion of methoxy groups into silanol groups. These results indicate that hydrolysis modified the trimethoxysilane moiety while retaining the methacrylate functionality required for subsequent photopolymerization.

### 2.2. Morphological Analysis

**Figure 3:**
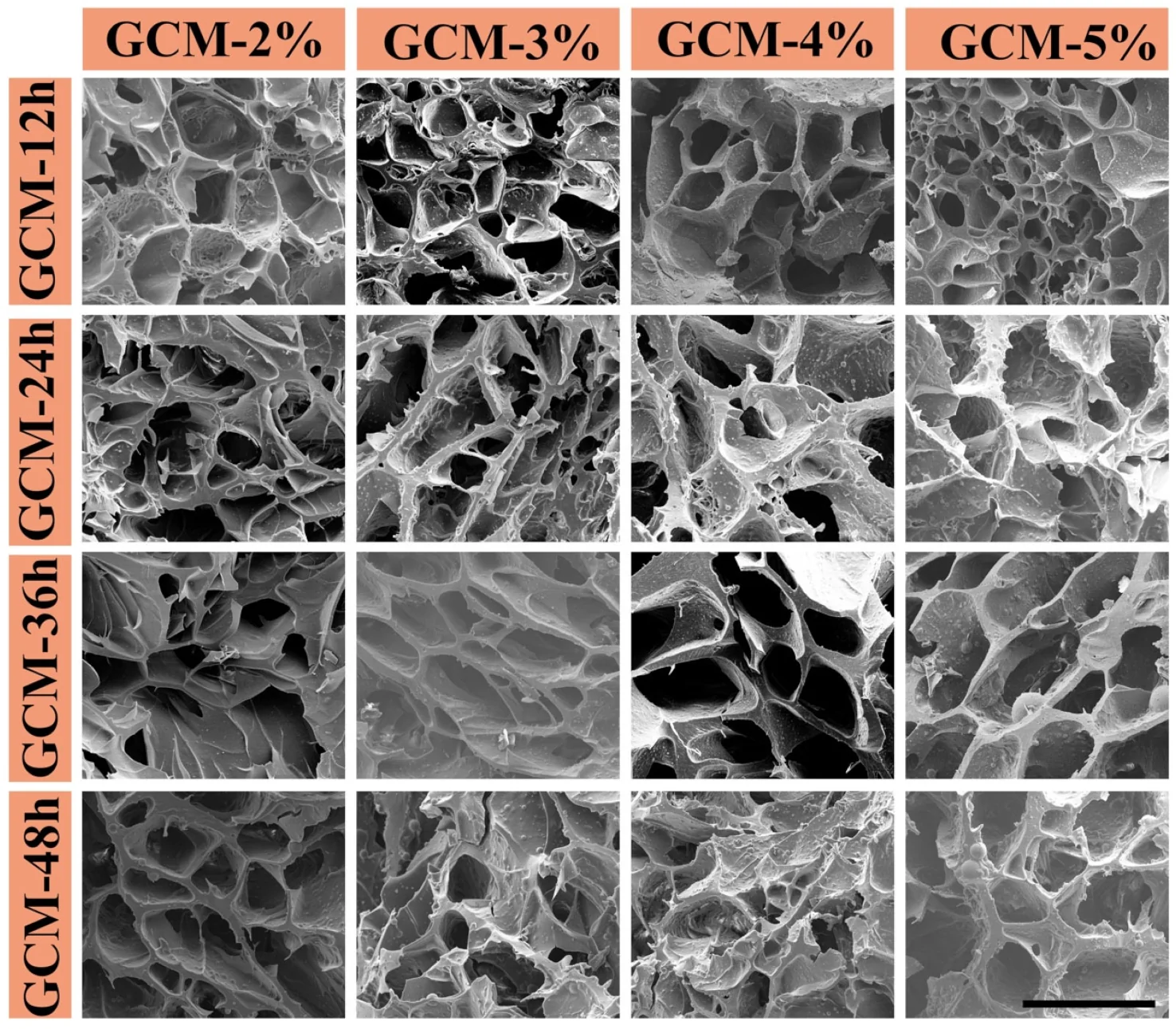
SEM images of freeze-dried GCM hydrogels prepared with different MPTS concentrations and prehydrolysis times. Scale bar = 300 μm, and the scale bar applies to all images.

To investigate the influence of the hydrolyzed MPTS sol on freeze-dried morphology, GCM hydrogels with different formulations were characterized by SEM, as shown in Figure 3. All samples exhibited interconnected porous morphologies. Compared with the smooth pore walls of the GelMA/CSMA hydrogel, the GCM hydrogels displayed numerous micrometer-scale granular features on the pore surfaces.

Increasing the MPTS concentration from 2% to 5% increased the apparent abundance of these granular features, whereas no marked change in macropore size was observed. Prolonging the hydrolysis time from 12 to 48 h resulted in larger and more pronounced granular structures. These observations suggest that MPTS concentration and hydrolysis time influence the formation and organization of MPTS-derived domains within the dried hydrogel microstructure. These features may be associated with the formation and reorganization of MPTS-derived siloxane species. These morphological changes may be associated with further condensation and reorganization of MPTS-derived siloxane species during prolonged hydrolysis. At a given MPTS concentration, hydrolysis time had a greater effect on the size of the granular features than on apparent abundance, whereas MPTS concentration predominantly affected their abundance, suggesting that the two parameters influenced the organization of MPTS-derived species in different ways.

### 2.3. XRD Analysis

**Figure 4:**
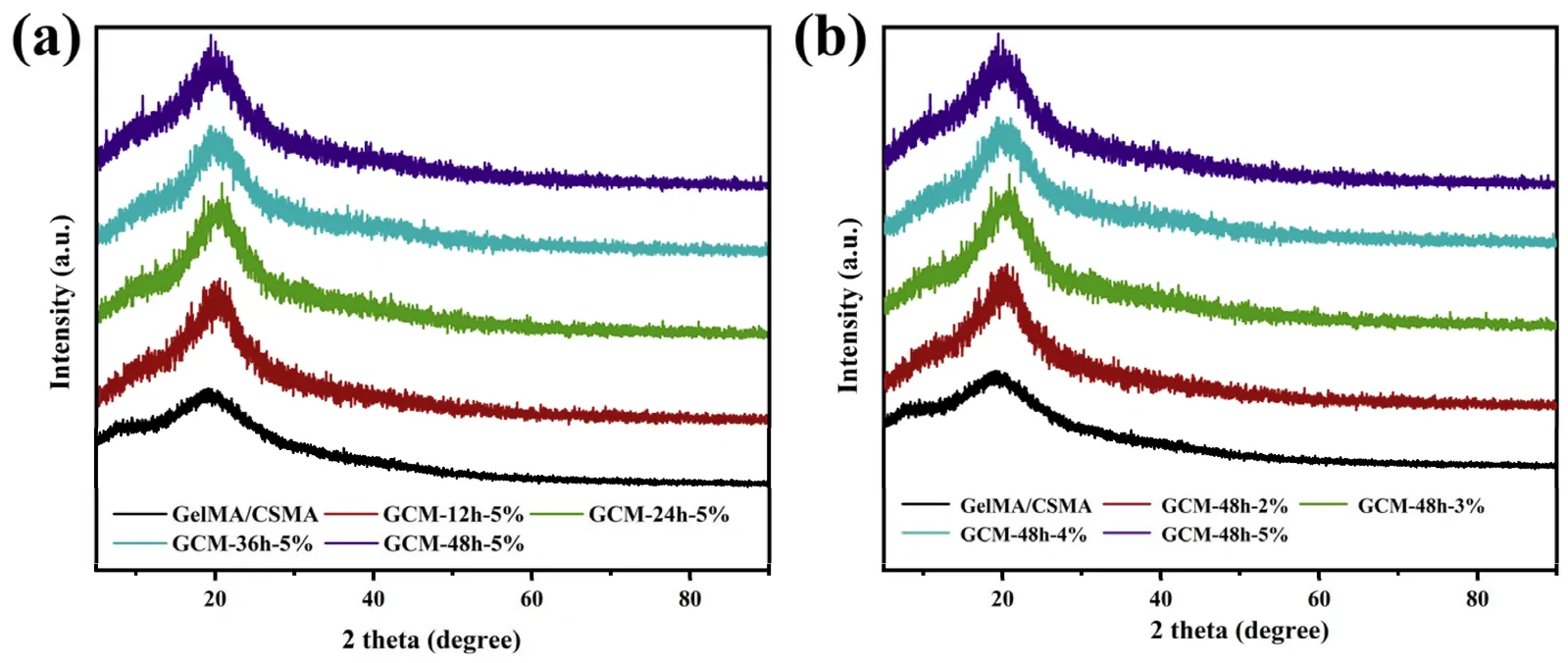
XRD patterns of GelMA/CSMA and GCM composite hydrogels: (**a**) GCM-5% samples prepared with different MPTS prehydrolysis times and (**b**) GCM-48h samples prepared with different MPTS concentrations.

To investigate the effect of MPTS hydrolysate incorporation on the crystalline structure of the hydrogels, XRD analysis was performed on the GCM composite hydrogels, and the results are shown in Figure 4. The XRD pattern of the GelMA/CSMA hydrogel exhibited two broad diffuse peaks at approximately 7.6° and 19.1°, consistent with the predominantly amorphous character commonly observed in gelatin- and GelMA-based materials [37]. Upon incorporation of MPTS hydrolysate, no additional sharp crystalline diffraction peaks were observed in the GCM hydrogels, indicating that the formed siloxane species were present in an amorphous state. With increasing MPTS concentration, the diffuse peak near 20° showed a slight shift to lower 2θ values, suggesting changes in the local short-range organization of the composite network. Similarly, prolonging the hydrolysis time from 12 h to 48 h also resulted in a discernible shift in this peak, suggesting changes in the local structural organization of the siloxane species within the polymer matrix. The shift in the diffuse XRD peak correlated well with the SEM observations (Figure 3), where the number and size of granular protrusions on the pore walls increased with increasing MPTS concentration and hydrolysis time. The absence of additional sharp diffraction peaks suggests that the MPTS-derived phase was predominantly amorphous. The variation in the broad diffraction feature with MPTS concentration and prehydrolysis time suggests changes in the local organization of the composite network. Together with the SEM observations, these results indicate that MPTS prehydrolysis conditions influenced the structural organization of the resulting GCM hydrogels.

### 2.4. Rheological Properties

**Figure 5:**
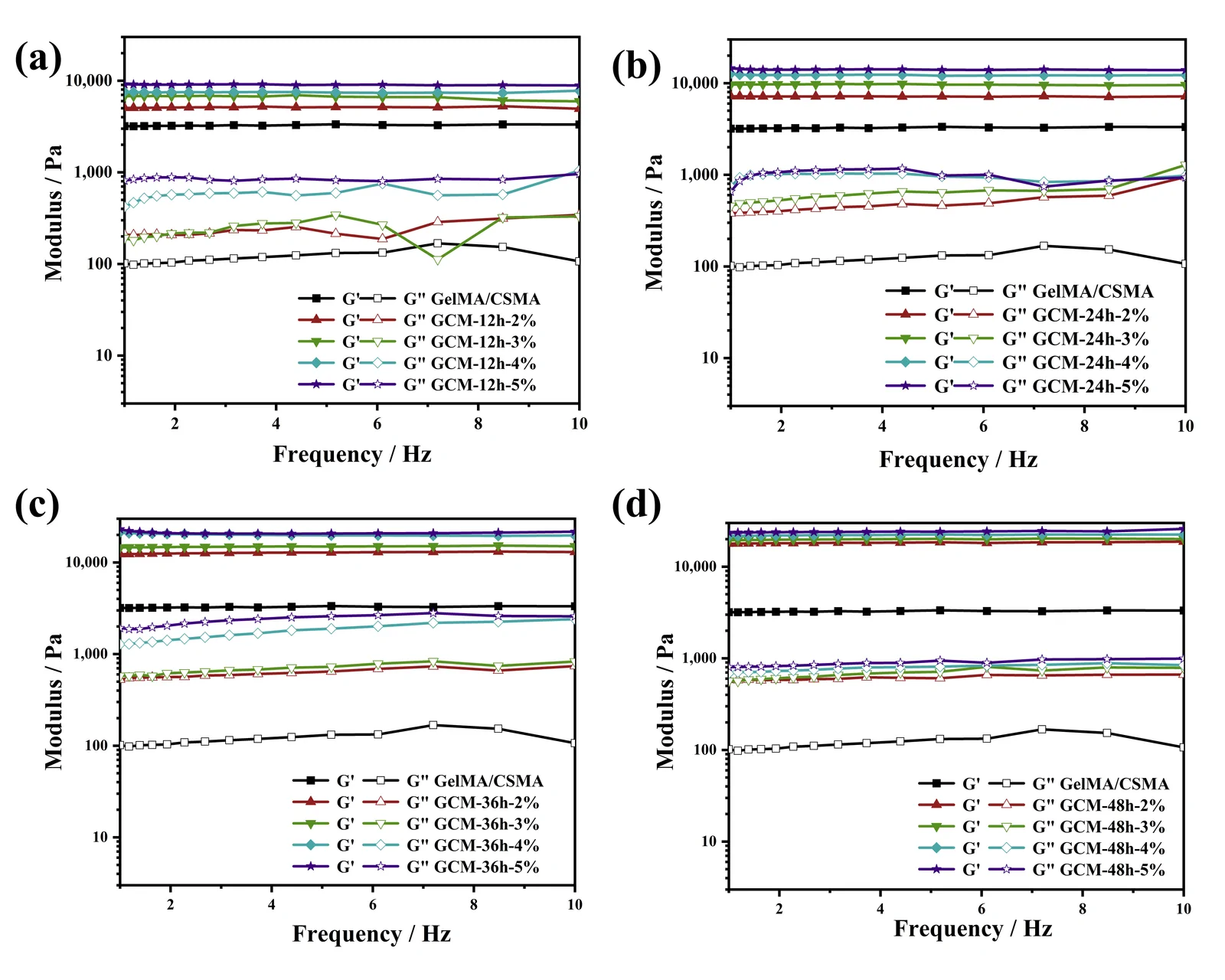
Frequency-dependent storage modulus (G′) and loss modulus (G″) of GCM hydrogels prepared with MPTS prehydrolysis times of (**a**) 12 h, (**b**) 24 h, (**c**) 36 h, and (**d**) 48 h.

As shown in Figure 5, the storage modulus (G′) of all hydrogel groups remained higher than the loss modulus (G″) throughout the tested frequency range, indicating that the photocrosslinked GCM hydrogels exhibited predominantly elastic responses and pronounced solid-like behavior. At the same prehydrolysis time, G′ generally increased with increasing MPTS concentration; at the same MPTS concentration, G′ also showed an overall increase with prolonged prehydrolysis time. These results indicate that increasing the MPTS concentration and extending the prehydrolysis time enhanced the network elasticity and structural stability of the hydrogels. As the MPTS concentration increased, the content of MPTS-derived siloxane structures in the system increased accordingly. The methacrylate groups retained on these structures may participate in photopolymerization with those of GelMA and CSMA to form covalent crosslinks, potentially increasing the number of covalent connections and effective network constraints [38,39]. Meanwhile, the silanol groups may contribute to interfacial interactions, including hydrogen bonding, with the hydroxyl, amino, and amide groups of GelMA and CSMA. These possible interactions, together with the presence of MPTS-derived siloxane structures, may restrict polymer-chain mobility and contribute to the observed increase in storage modulus.

### 2.5. Mechanical Properties

**Figure 6:**
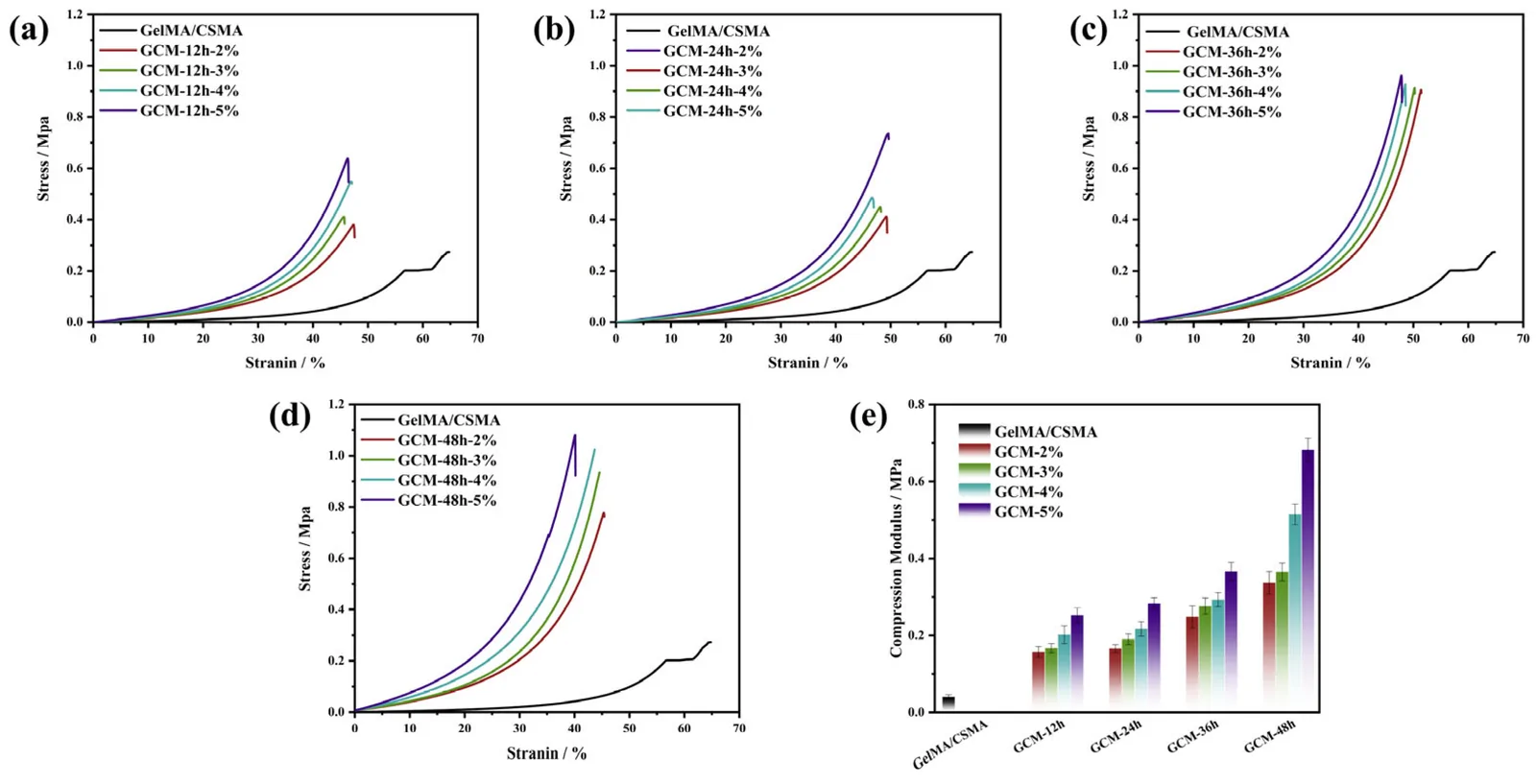
Compressive properties of GCM hydrogels: Stress–strain curves of samples prepared using MPTS prehydrolysis times of (**a**) 12 h, (**b**) 24 h, (**c**) 36 h, and (**d**) 48 h; (**e**) compressive moduli of the different GCM formulations.

Figure 6 presents the compressive stress–strain curves of the GCM hydrogels prepared with different MPTS concentrations and prehydrolysis times. The compressive modulus and maximum compressive stress values are summarized in Table 1 and Table 2, respectively. The GelMA/CSMA hydrogel without MPTS exhibited a compressive modulus of 0.040 MPa (Table 1). After the introduction of the MPTS-derived phase, the compressive modulus increased markedly, and all GCM formulations showed significantly higher values than the GelMA/CSMA control (*p* < 0.0001). Moreover, the GCM hydrogels exhibited clear concentration- and time-dependent responses. In particular, GCM-48h-5% achieved a compressive modulus of 0.682 MPa and a maximum compressive stress of 1.081 MPa, corresponding to approximately 16.9- and 4.0-fold increases over the GelMA/CSMA control, respectively.

A similar enhancement was observed for the maximum compressive stress (Table 2). The GelMA/CSMA control exhibited a maximum compressive stress of 0.273 MPa, whereas all GCM formulations showed significantly higher values than the control (*p* < 0.0001). The maximum compressive stress generally increased with increasing MPTS concentration at the same prehydrolysis time, although the effect of prehydrolysis time was not strictly monotonic for all formulations. GCM-48h-5% exhibited the highest maximum compressive stress of 1.081 MPa, approximately 4.0 times that of the GelMA/CSMA control.

The improvement in compressive performance may be associated with the incorporation of MPTS-derived siloxane structures and their interactions with the GelMA/CSMA network. First, the methacrylate groups retained on the MPTS-derived siloxane species may copolymerize with those of GelMA and CSMA during photocrosslinking, increasing the network crosslinking density and restricting relative polymer-chain slippage under compression [40]. Second, increasing the MPTS concentration increased the amount of MPTS-derived siloxane structures within the hydrogel. These relatively rigid structures may act as a reinforcing phase and contribute to load bearing under compression [25,41]. Meanwhile, silanol groups may form interfacial interactions, including hydrogen bonding, with the hydroxyl, amino, and amide groups of GelMA and CSMA, which may strengthen the association between the siloxane structures and the polymer network and facilitate load transfer. Prolonged prehydrolysis may further promote the condensation and structural reorganization of the MPTS-derived species, potentially leading to more pronounced siloxane reinforcing structures and greater resistance of the pore walls to compressive deformation.

### 2.6. Swelling Properties

**Figure 7:**
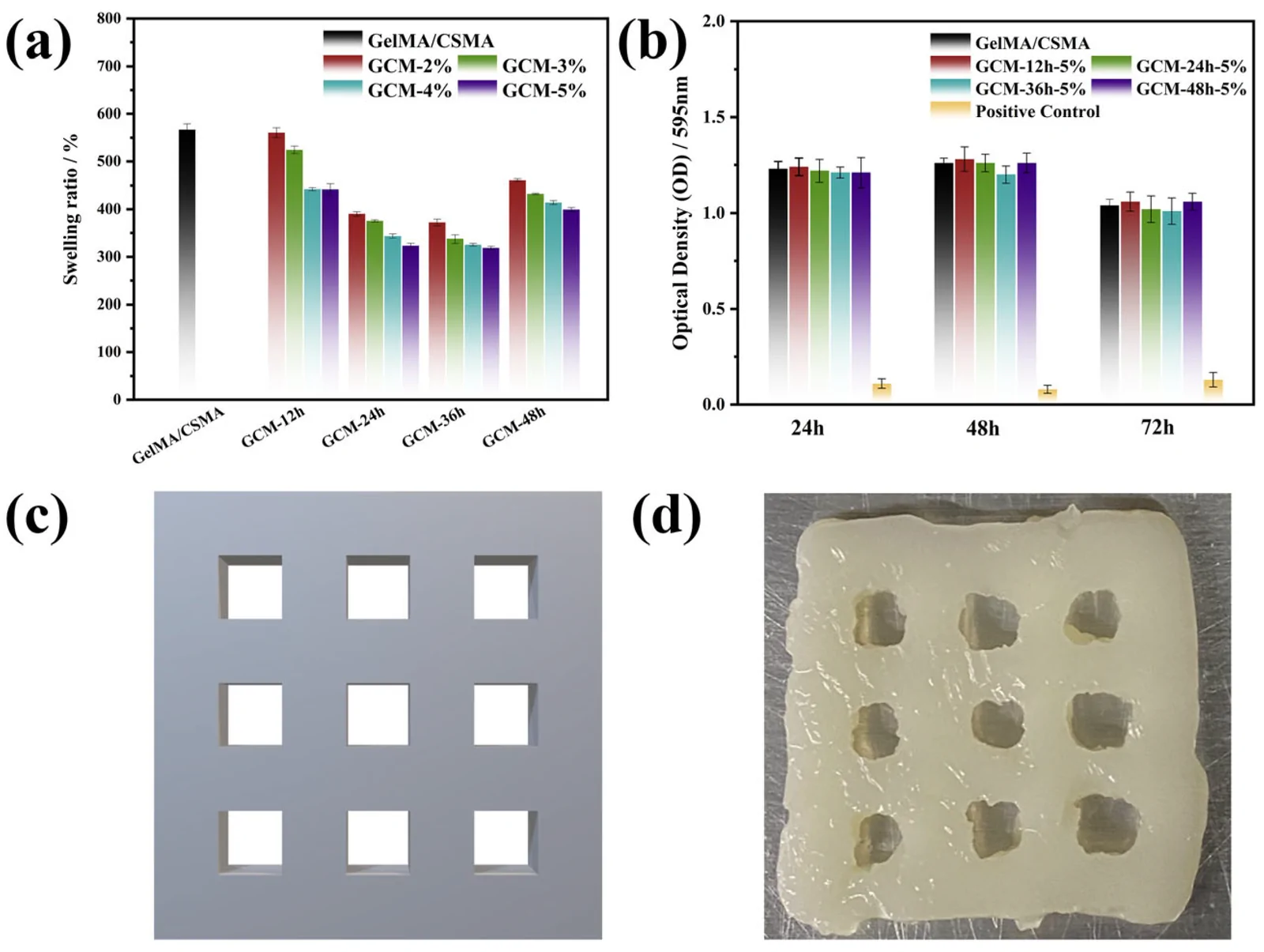
Swelling behavior, cytocompatibility, and printing feasibility of GCM hydrogels: (**a**) 24 h swelling ratios of GCM hydrogels prepared using different MPTS precursor concentrations and prehydrolysis times; (**b**) MTT absorbance of L929 cells cultured with extracts of selected GCM hydrogels for 24, 48, and 72 h; (**c**) CAD model of the porous plate containing a 3 × 3 square-pore array; and (**d**) photograph of the printed GCM-48h-5% hydrogel structure.

The swelling behavior of hydrogels reflects the balance between water uptake and structural stability in hydrated environments. Moderate water absorption facilitates body-fluid penetration and mass transport, whereas excessive swelling may cause network expansion, pore-structure deformation, and deterioration of mechanical properties. As shown in Figure 7a and Table 3, the GelMA/CSMA hydrogel without MPTS exhibited a swelling ratio of 566.94 ± 12.23%. Compared with the GelMA/CSMA control, the swelling ratios of all GCM formulations except GCM-12h-2% were significantly reduced (*p* < 0.0001), whereas no significant difference was observed for GCM-12h-2% (*p* > 0.05). At the same prehydrolysis time, the swelling ratio generally decreased with increasing MPTS concentration, indicating that the incorporation of the MPTS-derived phase improved the swelling resistance of the composite hydrogels. The reduced swelling ratio may be associated with the additional network constraints introduced by the MPTS-derived siloxane structures and their interactions with the GelMA/CSMA matrix, which may restrict polymer-chain expansion during hydration. Unlike the generally monotonic effect of MPTS concentration, the influence of prehydrolysis time on the swelling ratio was non-monotonic. The swelling ratio decreased as the prehydrolysis time increased from 12 to 36 h, with GCM-36h-5% exhibiting the lowest value, but partially increased when the prehydrolysis time was further extended to 48 h. This behavior may be related to changes in the organization of the MPTS-derived species during prolonged prehydrolysis. Further condensation and structural reorganization may contribute to greater constraints on polymer-chain expansion, whereas the formation of larger granular structures at 48 h, as suggested by the SEM observations, could increase structural heterogeneity and reduce effective polymer–siloxane contact in some regions, thereby allowing greater hydration and polymer-chain expansion.

### 2.7. Biocompatibility

The in vitro cytocompatibility of GCM hydrogels was evaluated using MTT assays. Before biological evaluation, the hydrogel tablets were immersed in 50 mL of ethanol for 7 days to remove residual small molecules, including lithium phenyl (2,4,6-trimethylbenzoyl) phosphinate (LAP), and subsequently sterilized by autoclaving. L929 cells were cultured with extracts obtained from the different GCM hydrogel formulations. MPTS-free GelMA/CSMA hydrogel was used as the material control, while medium containing 0.64% phenol served as the positive cytotoxicity control. As shown in Figure 7b and Table 4, no significant differences in MTT absorbance were observed between the GCM formulations and the GelMA/CSMA material control at 24, 48, or 72 h (*p* > 0.05). In contrast, the positive cytotoxicity control exhibited markedly lower MTT absorbance (*p* < 0.0001). These results suggest that incorporation of the MPTS-derived phase did not adversely affect L929 cellular metabolic activity under the tested extract conditions.

### 2.8. DLP Printing Performance

To explore the potential of GCM hydrogels for DLP 3D printing, the hydrogel precursor containing GelMA, CSMA, MPTS hydrolysate, and LAP was used to fabricate a porous plate containing a 3 × 3 square-pore array using a DLP printer equipped with a 405 nm LED light source. The printing layer thickness was set to 50 μm, with a light intensity of 80 mW cm^−2^ and an exposure time of 60 s per layer. As shown in Figure 7c,d, the overall pore-array geometry was qualitatively reproduced in the printed construct, demonstrating the feasibility of layer-by-layer fabrication. These results demonstrate that the GCM formulation was compatible with photocuring and layer-by-layer formation under the tested printing conditions. Together with its enhanced mechanical properties and favorable cytocompatibility, these findings support the potential of GCM hydrogels for DLP fabrication of customized three-dimensional scaffolds for further tissue-engineering applications.

## 3. Conclusions

In conclusion, GCM composite hydrogels were successfully prepared by integrating MPTS-derived siloxane formation with the photocrosslinking of GelMA and CSMA. MPTS concentration and prehydrolysis time were associated with changes in the structural organization, mechanical performance, and swelling behavior of the hydrogels. Increasing MPTS concentration generally enhanced the storage modulus and compressive properties while reducing swelling, whereas variation in prehydrolysis time produced more complex effects on mechanical reinforcement and swelling behavior. Among the tested formulations, GCM-48h-5% exhibited markedly improved mechanical properties, together with demonstrated DLP printing feasibility and no detectable adverse effect on L929 cellular metabolic activity under the tested extract conditions. These findings demonstrate a molecular-precursor strategy for introducing MPTS-derived siloxane reinforcing structures into photocrosslinked biopolymer hydrogels without relying on separately synthesized and dispersed silica nanoparticles and support the potential of this approach for DLP-fabricated tissue-engineering scaffolds. Future studies may further explore the degradation behavior and long-term structural stability of GCM hydrogels under physiologically relevant conditions, together with their long-term biocompatibility and in vivo performance. Continued optimization of MPTS prehydrolysis, hydrogel preparation, and DLP fabrication may further improve the reliability and reproducibility of the system and support future scale-up. In addition, consideration of manufacturing costs and regulatory requirements will be important for further evaluating the translational potential of GCM hydrogels in biomedical applications.

## 4. Materials and Methods

### 4.1. Materials

Gelatin, glacial acetic acid, glycidyl methacrylate (GMA), methacrylic anhydride (MAA), and sodium hydroxide were purchased from Macklin Biochemical Technology Co., Ltd. (Shanghai, China). Chitosan was purchased from Aladdin Biochemical Technology Co., Ltd. (Shanghai, China). PBS buffer was obtained from Bairuiji Biotechnology Co., Ltd. (Beijing, China). Phenyl(2,4,6-trimethylbenzoyl) phosphinate (LAP) was provided by Bide Pharmaceutical (Shanghai, China). γ-Methacryloxypropyl trimethoxysilane (MPTS) was sourced from Shanyi Plastic Chemical Co., Ltd. (Dongguan, China). Deionized water was prepared in our laboratory. All other reagents were of analytical grade and used as received.

### 4.2. Preparation of GelMA

GelMA was synthesized according to previously published protocol with minor modifications [42]. Briefly, gelatin (50 g) was mixed with PBS (500 mL) in a 1000 mL three-neck flask under continuous stirring at 50 °C. After complete dissolution, GMA (50 mL) was added dropwise to the reaction flask over 30 min. The reaction was allowed to proceed at 50 °C for 10 h. The resulting solution was then dialyzed against deionized water using 8–14 kDa cutoff dialysis membrane tubing for 4 days with frequent water changes to eliminate unreacted monomers and impurities. Finally, the obtained dialyzed aqueous solution was lyophilized to obtain yellow solid GelMA.

### 4.3. Preparation of CSMA

CSMA was synthesized according to previously published protocol with minor modifications [43]. Briefly, chitosan (CS, 5 g) was dissolved in 1 wt% aqueous acetic acid solution (500 mL) in a 1000 mL three-necked flask under stirring at 60 °C. After complete dissolution, the solution was cooled to room temperature (25 °C). MAA (11.46 g) was added dropwise into the solution, and the mixture was stirred for 24 h. The crude product was diluted with deionized water and dialyzed against deionized water using dialysis membranes (8–14 kDa) for 4 days with frequent water changes. The purified solution was lyophilized to obtain a white solid product.

### 4.4. Preparation of MPTS Precursor Solutions

Aqueous MPTS precursor solutions with concentrations of 2%, 3%, 4%, and 5% (*w*/*v*) were prepared by adding 2, 3, 4, and 5 g of MPTS, respectively, and adjusting the final volume of each solution to 100 mL with deionized water. The MPTS concentration range of 2–5% (*w*/*v*) was selected based on preliminary formulation observations, as higher MPTS concentrations led to visible sedimentation and reduced precursor stability. The solutions were continuously stirred at 50 °C for 12, 24, 36, and 48 h, respectively, to allow MPTS hydrolysis and condensation. The resulting precursor solutions were subsequently used for the preparation of GCM hydrogels.

### 4.5. Preparation of GCM Hydrogels

GCM composite hydrogels were prepared by photopolymerization. For each formulation, 20 g of GelMA and 0.5 g of CSMA were sequentially added to 100 mL of the corresponding prehydrolyzed MPTS precursor solution and stirred for 3 h and 2 h, respectively, until completely dissolved. LAP was subsequently added at 0.5 wt% relative to the mass of GelMA under dark conditions and stirred for 10 min. The resulting precursor solution was degassed under vacuum to remove air bubbles, transferred into molds, and irradiated at 405 nm for 8 min to obtain the GCM hydrogels. The detailed compositions are summarized in Table 5.

### 4.6. Structural Characterization

The chemical structures of GelMA, CSMA and siloxane species were confirmed by FTIR spectroscopy (iN10MX, Thermo Scientific. Waltham, MA, USA) in the range of 4000–400 cm^−1^ using KBr pellets. ^1^H NMR spectra were recorded on a Bruker AVANCE III 400 MHz spectrometer (Bruker Corporation, Billerica, MA, USA) using deuterium oxide (D_2_O) as the solvent.

### 4.7. Morphological Characterization

The surface morphology of the freeze-dried hydrogels was observed using a scanning electron microscope (SEM, TESCAN MIRA LMS, TESCAN ORSAY HOLDING, a.s., Brno, Czech Republic). Samples were freeze-dried, fractured, and sputter-coated with gold prior to imaging. The accelerating voltage was 5 kV.

### 4.8. XRD Characterization

The crystallinity of GelMA/CSMA and GCM hydrogels was examined by X-ray diffraction analysis. An X-ray diffractometer with Cu Kα radiation (λ = 0.154 nm), a 40 kV voltage, and a 40 mA current (Rigaku D/Max2500VB2+/Pc diffractometer, Rigaku Company, Tokyo, Japan), was used. The scanning range was from 5° to 90°, with a scanning speed of 5°/min.

### 4.9. Rheological Characterization

Rheological properties of the GCM hydrogels were measured using a HAAKE MARS rheometer (Thermo Fisher Scientific, Waltham, MA, USA) equipped with a parallel-plate geometry (diameter, 20 mm, gap, 1 mm). Disc-shaped samples (thickness 1.2 mm, diameter 20 mm) were used. Frequency sweep tests were performed at 30 °C within the linear viscoelastic region (determined by strain sweep at 1 Hz) at a constant strain of 0.5%, with frequency ranging from 0.1 to 10 Hz. A solvent trap was applied to prevent water evaporation. For each formulation, five independent samples were tested, and the storage modulus (G′) and loss modulus (G″) were recorded.

### 4.10. Mechanical Property Characterization

Compressive mechanical properties of the as prepared GCM samples were evaluated using a CMT5305 universal testing machine (MTS Systems (China) Co., Ltd., Shenzhen, China). Cylindrical specimens with a diameter of 10 mm and a height of 5 mm were prepared. The compression test was performed at a crosshead speed of 10 mm/min until fracture. The compressive modulus was calculated from the initial linear region of the stress–strain curve (strain 0–15%). Five parallel samples were tested for each formulation.

### 4.11. Swelling Properties Test

The swelling behavior of the GCM hydrogels was quantitatively evaluated using the swelling ratio (*SR*). The samples were first freeze-dried to obtain their dry weights (*W*_0_). Subsequently, the dried samples were immersed in deionized water for 24 h. The swollen samples were then carefully removed, and the surface water was gently blotted with filter paper before measuring the wet weights (*W_t_*). The swelling ratio (*SR*) was then calculated according to Equation (1).

(1) $$SR=\frac{W_{t}-W_{0}}{W_{0}}\times 100\%$$

All measurements were carried out on five independent parallel samples (*n* = 5) for each formulation.

### 4.12. DLP Printing Performance

3D printing was conducted on an ANYCUBIC Photon Mono 4K printer (405 nm, 80 mW·cm^−2^) with a layer thickness of 50 μm and an exposure time of 60 s per layer. Printed constructs were rinsed with deionized water and post-cured under UV.

### 4.13. Biocompatibility Characterization

The cytocompatibility of GCM was evaluated by the MTT assay. The hydrogel specimens were immersed in 50 mL of ethanol for 7 days to remove residual small molecules. Sterilized hydrogel samples were immersed in DMEM for 12 h to prepare the extracts. The extracts were then added to 96-well plates seeded with L929 cells and incubated for 24 h, 48 h, and 72 h, respectively. MPTS-free GelMA/CSMA hydrogel was used as the material control, whereas medium containing 0.64% phenol was used as the positive cytotoxicity control. After incubation, 20 μL of MTT solution was added to each well, and the 96 well plates were incubated at 37 °C in a 5% CO_2_ atmosphere for 4 h. Following incubation, dimethyl sulfoxide (DMSO) was added to dissolve the purple formazan crystals, and the optical density (OD) was measured at 595 nm. The measured OD values were used as the quantitative readout of the MTT assay.

### 4.14. Statistical Analysis

All quantitative data are presented as the mean ± standard deviation (SD) (n = 5). For compressive modulus, maximum compressive stress, swelling ratio, and MTT measurements, intergroup differences were assessed using one-way analysis of variance (ANOVA), followed by Dunnett’s post hoc test to compare each GCM formulation with the GelMA/CSMA material control. For the MTT assay, statistical analyses were performed separately at each incubation time point. Significance levels are indicated as follows: *p* < 0.05 (*), *p* < 0.01 (**), *p* < 0.001 (***), and *p* < 0.0001 (****); *p* ≥ 0.05 was considered not significant (ns).

## Figures and Tables

**Table 1:** Compressive modulus of GelMA/CSMA and GCM hydrogels with different MPTS precursor concentrations and prehydrolysis times.

Sample Code	Compression Modulus/MPa				
	Mean ± Standard Deviation/MPa				
	0%	2%	3%	4%	5%
GelMA/CSMA	0.040 ± 0.006				
GCM-12h		0.157 ± 0.014 ***	0.167 ± 0.012 ***	0.202 ± 0.023 ***	0.252 ± 0.019 ***
GCM-24h		0.166 ± 0.010 ***	0.190 ± 0.014 ***	0.217 ± 0.019 ***	0.283 ± 0.015 ***
GCM-36h		0.248 ± 0.029 ***	0.276 ± 0.021 ***	0.293 ± 0.018 ***	0.366 ± 0.024 ***
GCM-48h		0.337 ± 0.029 ***	0.365 ± 0.023 ***	0.514 ± 0.027 ***	0.682 ± 0.031 ***

GCM-12h, GCM-24h, GCM-36h, and GCM-48h showed a statistically significant difference from GelMA/CSMA. *** *p* < 0.001.

**Table 2:** Maximum compressive stress of GelMA/CSMA and GCM hydrogels with different MPTS precursor concentrations and prehydrolysis times.

Sample Code	Maximum Compressive Stress/MPa				
	Mean ± Standard Deviation/MPa				
	0%	2%	3%	4%	5%
GelMA/CSMA	0.273 ± 0.022				
GCM-12h		0.380 ± 0.015 **	0.411 ± 0.023 ***	0.548 ± 0.031 ***	0.639 ± 0.057 ***
GCM-24h		0.410 ± 0.038 ***	0.450 ± 0.019 ***	0.485 ± 0.043 ***	0.736 ± 0.026 ***
GCM-36h		0.906 ± 0.056 ***	0.914 ± 0.061 ***	0.928 ± 0.053 ***	0.962 ± 0.047 ***
GCM-48h		0.777 ± 0.037 ***	0.934 ± 0.031 ***	1.024 ± 0.042 ***	1.081 ± 0.048 ***

GCM-12h, GCM-24h, GCM-36h, and GCM-48h showed a statistically significant difference from GelMA/CSMA. ** *p* < 0.01, *** *p* < 0.001.

**Table 3:** The swelling ratio of GCM samples.

Sample Code	Swelling Ratio/%				
	Mean ± Standard Deviation/%				
	0%	2%	3%	4%	5%
GelMA/CSMA	566.94293 ± 12.23				
GCM-12h		560.66 ± 10.19 ^ns^	524.23 ± 7.81 ***	442.31 ± 3.24 ***	441.96 ± 11.78 ***
GCM-24h		390.17 ± 4.72 ***	375.40 ± 2.25 ***	343.26 ± 4.70 ***	323.46 ± 5.29 ***
GCM-36h		372.19 ± 7.07 ***	337.13 ± 8.89 ***	325.58 ± 2.90 ***	318.79 ± 3.56 ***
GCM-48h		461.19 ± 2.35 ***	432.31 ± 1.31 ***	414.03 ± 4.06 ***	399.37 ± 4.18 ***

GCM-12h, GCM-24h, GCM-36h, and GCM-48h showed a statistically significant difference from GelMA/CSMA. *** *p* < 0.001; ns, not significant.

**Table 4:** MTT absorbance (OD) values of L929 cells cultured with extracts of GCM samples.

Sample Code	OD Values (Mean ± Standard Deviation)		
	24 h	48 h	72 h
GelMA/CSMA	1.23 ± 0.038	1.28 ± 0.046	1.04 ± 0.031
GCM-12h-5%	1.24 ± 0.046 ^ns^	1.24 ± 0.045 ^ns^	1.06 ± 0.049 ^ns^
GCM-24h-5%	1.22 ± 0.058 ^ns^	1.26 ± 0.058 ^ns^	1.02 ± 0.070 ^ns^
GCM-36h-5%	1.21 ± 0.029 ^ns^	1.20 ± 0.029 ^ns^	1.01 ± 0.069 ^ns^
GCM-48h-5%	1.21 ± 0.078 ^ns^	1.26 ± 0.078 ^ns^	1.06 ± 0.043 ^ns^
Positive Control	0.11 ± 0.024 ***	0.08 ± 0.043 ***	0.13 ± 0.038 ***

GCM-12h, GCM-24h, GCM-36h, and GCM-48h showed a statistically significant difference from the positive control, while showed no significant difference from GelMA/CSMA. *** *p* < 0.001; ns, not significant.

**Table 5:** Compositions of the GelMA/CSMA and GCM hydrogel precursor formulations.

Sample Code		MPTS Precursor Concentration (% *w*/*v*)	GelMA (g/100 mL Precursor)	CSMA (g/100 mL Precursor)	LAP	Prehydrolysis Time (h)
Control	GelMA/CSMA	0	20	0.5	0.5 wt% of GelMA	0
Hydrolyzed for 12 h	GCM-12h-2%	2	20	0.5	0.5 wt% of GelMA	12
	GCM-12h-3%	3	20	0.5	0.5 wt% of GelMA	12
	GCM-12h-4%	4	20	0.5	0.5 wt% of GelMA	12
	GCM-12h-5%	5	20	0.5	0.5 wt% of GelMA	12
Hydrolyzed for 24 h	GCM-24h-2%	2	20	0.5	0.5 wt% of GelMA	24
	GCM-24h-3%	3	20	0.5	0.5 wt% of GelMA	24
	GCM-24h-4%	4	20	0.5	0.5 wt% of GelMA	24
	GCM-24h-5%	5	20	0.5	0.5 wt% of GelMA	24
Hydrolyzed for 36 h	GCM-36h-2%	2	20	0.5	0.5 wt% of GelMA	36
	GCM-36h-3%	3	20	0.5	0.5 wt% of GelMA	36
	GCM-36h-4%	4	20	0.5	0.5 wt% of GelMA	36
	GCM-36h-5%	5	20	0.5	0.5 wt% of GelMA	36
Hydrolyzed for 48 h	GCM-48h-2%	2	20	0.5	0.5 wt% of GelMA	48
	GCM-48h-3%	3	20	0.5	0.5 wt% of GelMA	48
	GCM-48h-4%	4	20	0.5	0.5 wt% of GelMA	48
	GCM-48h-5%	5	20	0.5	0.5 wt% of GelMA	48

The amounts of GelMA and CSMA are expressed relative to 100 mL of the corresponding MPTS precursor solution. LAP was added at 0.5 wt% relative to the mass of GelMA.

## Data Availability

The original contributions presented in this study are included in the article. Further inquiries can be directed to the corresponding author(s).

## Abbreviations

The following abbreviations are used in this manuscript:

GelMA	Gelatin methacryloyl
CSMA	Methacrylated chitosan
MPTS	γ-Methacryloxypropyltrimethoxysilane
GCM	GelMA/CSMA/MPTS composite hydrogel
LAP	Lithium phenyl-2,4,6-trimethylbenzoylphosphinate
DLP	Digital Light Processing
FTIR	Fourier-transform infrared spectroscopy
SEM	Scanning electron microscopy
XRD	X-ray diffraction
MTT	3-(4,5-dimethylthiazol-2-yl)-2,5-diphenyltetrazolium bromide
